# Distal versus proximal radial access in coronary angiography: a meta-analysis

**DOI:** 10.1007/s00392-024-02505-3

**Published:** 2024-09-17

**Authors:** Julia Lueg, Daniel Schulze, Robert Stöhr, David M. Leistner

**Affiliations:** 1 Deutsches Herzzentrum der Charité, Klinik für Kardiologie und Angiologie, Department of Cardiology, Angiology and Intensive Care Medicine, Campus Mitte, Charitéplatz 1, 10117 Berlin, Germany; 2 Charité – Universitätsmedizin Berlin, corporate member of Freie Universität Berlin and Humboldt- Universität zu Berlin, Charitéplatz 1, 10117 Berlin, Germany; 3 DZHK (German Centre for Cardiovascular Research), partner site Berlin, Berlin, Germany; 4 Institute of Biometry and Clinical Epidemiology, Charité University Medicine, Campus Mitte, Berlin, Germany; 5 Department of Medicine, Cardiology, Goethe University Hospital, Frankfurt, Germany; 6 German Center for Cardiovascular Research (DZHK) Partner Site RheinMain, Frankfurt, Germany

**Keywords:** Distal radial access, Proximal radial access, Coronary angiography, Percutaneous coronary intervention, Radial arterial occlusion, Access failure

## Abstract

**Background:**

Distal radial access (DRA) represents a promising alternative to conventional proximal radial access (PRA) for coronary angiography. Substantial advantages regarding safety and efficacy have been suggested for DRA, but the ideal access route remains controversial.

**Aims:**

The aim of this study was to compare safety, efficacy and feasibility of DRA to PRA.

**Methods:**

National Library of Medicine PubMed, Web of Science, clinicaltrials.gov and Cochrane Library were systematically searched for randomized controlled trials and registry studies comparing DRA and PRA that were published between January 1, 2017 and April, 2024. Primary endpoint was the rate of radial artery occlusion (RAO). Secondary endpoints were access failure, access time, procedure time, arterial spasm, hematoma, and hemostasis time. Data extraction was performed by two independent investigators. Relative risks were aggregated using a random effects model. We applied meta-analytic regression to assess study characteristic variables as possible moderators of the study effects.

**Results:**

44 studies with a total of 21,081 patients were included. We found a significantly lower rate of RAO after DRA (DRA 1.28%, PRA 4.76%, *p* < .001) with a 2.92 times lower risk compared to the proximal approach (Log Risk Ratio = −1.07, *p* < .001). Conversely, the risk for access failure was 2.42 times higher for DRA compared to PRA (Log Risk Ratio = 0.88, *p* < .001).

**Conclusion:**

In this largest meta-analysis to date, we were able to show that rates of RAO are reduced with DRA compared to conventional PRA. This suggests DRA is a safe alternative to PRA.

**Graphical abstract:**

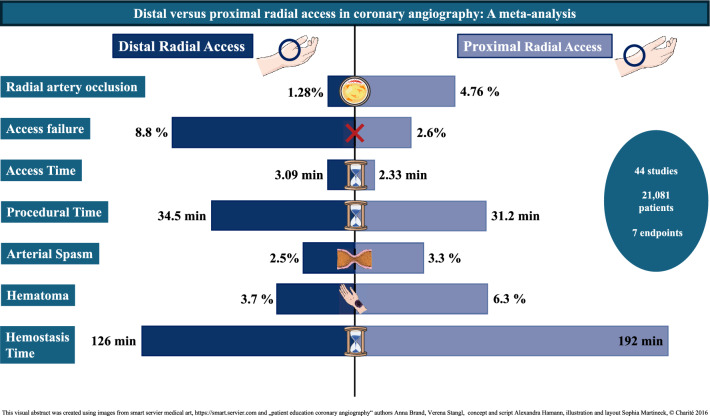

**Supplementary Information:**

The online version contains supplementary material available at 10.1007/s00392-024-02505-3.

## Introduction

Proximal radial access (PRA) is the guideline-recommended standard access strategy for coronary angiography (CAG) and percutaneous coronary intervention (PCI) [1]. Although relatively uncommon (occurring in 1–10% of cases), severe local vascular complications may arise during PRA [2]. Among these complications, radial arterial occlusion (RAO) is of particular importance, as it precludes repeat radial access and the use of the radial artery for arterioarterial coronary bypass and the creation of arteriovenous dialysis shunts [3]. To mitigate the risk of RAO and other access site complications, distal radial access (DRA) via the anatomical snuffbox has been proposed as an alternative to PRA [4–6]. Several smaller studies that compare the procedural data and outcomes of DRA and PRA have yielded mixed results [7–9]. The majority indicate that DRA is more beneficial than PRA in the prevention of local vascular complications, such as arterial spasm or hematoma. [7, 8]. The data on technical success rates is heterogeneous and spans a large percentage range [10, 11]. In order to provide a foundation for the development of standardized recommendations, a comprehensive and detailed examination of the existing data on the performance of DRA in comparison to PRA is necessary. This meta-analysis aims to integrate all literature comparing DRA and PRA access strategies published since the proposal of DRA in 2017 to systematically evaluate the safety and technical feasibility of DRA as a possible new standard access strategy for coronary angiography.

## Methods

### Search strategy

All studies comparing DRA and PRA published between January, 2017 and April, 2024 were included. This analysis was conducted in accordance with the Preferred Reporting Items for Systematic Review and Meta-Analyses (PRISMA) statement [12]. The National Library of Medicine PubMed, Web of Science, clinicaltrials.gov and Cochrane Library databases were used for literature research. Slightly different search strings were used for each database based on their syntax requirements. The following search string was used for PubMed and Cochrane Library: “((percutaneous coronary intervention OR coronary angiography OR coronary intervention) AND (distal radial access OR distal radial approach OR snuffbox)) AND (radial artery occlusion OR arterial spasm OR hemostasis)”. For Clinicaltrials.gov we used: “[condition or disease: (percutaneous coronary intervention OR coronary angiography OR coronary intervention) other terms: (distal radial access OR distal radial approach OR snuffbox OR radial artery occlusion)]” and for Web of Science: “((TS = ((percutaneous coronary intervention OR coronary angiography OR coronary intervention))) AND TS = ((distal radial access OR distal radial approach OR snuffbox))) AND TS = ((radial artery occlusion OR arterial spasm OR hemostasis))”.

### Data extraction

The inclusion criteria for this meta-analysis were (1) randomized controlled trials and registry studies, (2) investigation of safety and feasibility of DRA vs PRA, (3) studies that included the endpoint RAO and (4) one of the following indicators: access success rate, arterial spasm, hematoma, time to hemostasis, time to access, procedure time and crossover rate. This search strategy yielded a total of 44 studies, of which 43 evaluated DRA for coronary angiography and one for superficial femoral artery interventions. Study characteristics (year, study design, number of included patients, observation period and country) were collected for all studies fulfilling the above criteria. Variables of interest included age and sex, cardiovascular risk factors (smoking, diabetes, dyslipidemia) and procedural characteristics including the type of intervention (CAG, PCI). Two independent investigators (JL and DS) critically assessed the full texts from all 44 studies. Intraclass correlation (ICC) was calculated as measure of rater agreement. Discrepancies were discussed and consensus was reached in all cases.

### Risk of bias

To assess the potential risk of bias, a bespoke tool was created by the authors, who agreed on its suitability. As the studies included were of diverse types (observational and randomized), no existing tool was found to be comprehensive in its coverage of the relevant aspects for the review. We decided to draw from two established sources: Cochrane Collaboration and Joanna Briggs Institute [13, 14]. The studies were evaluated according to the following criteria: transparency of inclusion criteria, sample description, group allocation (and protocol deviations), bias due to confounding and completeness of result reporting. Details on the rating criteria can be found in the supplements (Supplementary Table 1).

### Statistics

We performed a statistical analysis using “metafor” for meta-analysis in R [15]. All analyses were carried out for the whole set of studies as well as for the subset of RCTs only. For group comparison, risk ratios were calculated for binary event outcomes (RAO, access success, arterial spasm, hematoma). In cases where an event did not occur at all in a study group, we added a frequency of 0.5 to both groups to make calculations feasible. In order to increase the comprehensibility of the data presented, we used the log of risk ratios in the forest plots. The continuous outcomes hemostasis time, access time, and total procedural time were analyzed by their group mean difference (unstandardized, unit: minutes). Some studies reported median and range instead of mean and standard deviation for the measured times [16, 17]. In these instances, we employed the methodology for estimating the mean and standard deviation in large samples, as described by Hozo et al. [18]. All outcomes were aggregated using a meta-analytic random effects model with a restricted maximum-likelihood estimator and Knapp-Hartung type test statistics [19]. Heterogeneity was evaluated by means of estimating the variance of the true effects (τ^2^) as well as the proportion of this variance in the studies’ variance (I^2^). If applicable, publication bias was examined by a funnel plot with an additional test for bias by means of regressing the effect sizes on the standard errors. The level of statistical significance was set at p < 0.05. A meta-analytic regression was employed to assess several study characteristic variables as potential moderators of the study effects. The study characteristics included the mean age, proportions in sex, type of intervention, presence of diabetes and hyperlipidemia, smoking status, and the year when patient recruitment commenced. These variables were processed in two ways: (1) as overall study characteristics and (2) as the group differences in these variables. All figures were created using R.

## Results

**Fig. 1:**
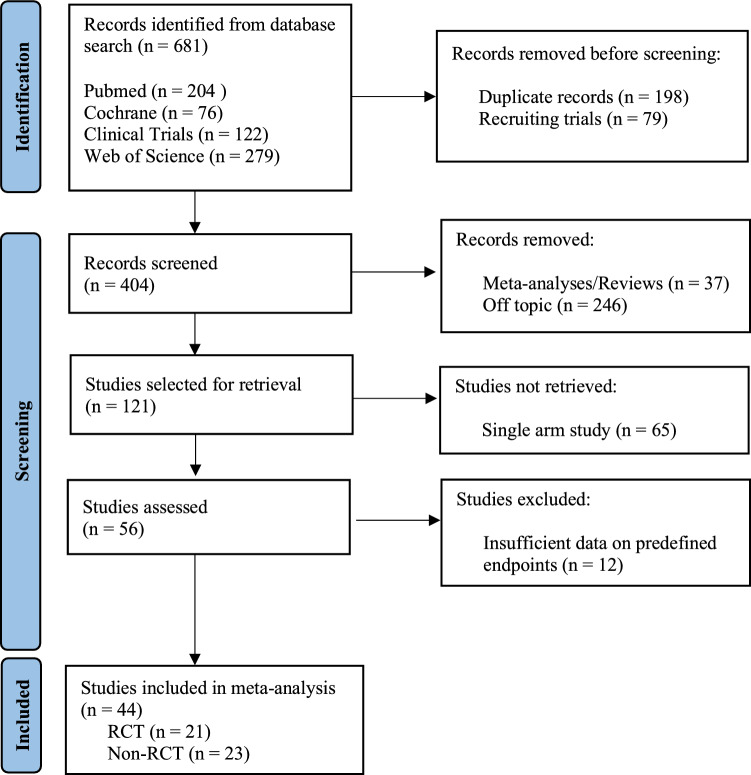
Literature search strategy based on the PRISMA statement

We identified 44 studies meeting the inclusion criteria, including a total of 21,081 patients. 21 studies were randomized controlled trials (RCT) and 23 studies were non-RCTs that provided data about safety and feasibility of DRA with a non-randomized control group (Fig. 1).

### Data extraction

Initial data extraction yielded a moderate inter-rater agreement for the primary outcome RAO (distal: ICC = 0.65, proximal: ICC = 0.62) and high agreements for the secondary outcomes (ICCs > 0.95). The main disparity for RAO existed between the studies by Kaledin [20], Li [16] and Zhang et al. [21] who assessed RAO in a subgroup of the patients with proximal access only. In order to maintain proportionality, we extrapolated the respective findings to the entire group.

### Baseline characteristics

Of a total of 21,081 patients, 10,399 were in the DRA- and 10,682 in the PRA-group. There were no significant differences between the groups in baseline characteristics (p > 0.591). However, studies varied substantially in the average patient characteristics (see Table 1). Mean ages ranged from 50 to 82 years, the proportion of male patients from 32 to 84%.

**Table 1:** Baseline characteristics

Study	N		Age		Male gender		PCI		DM		HLP		Smoking	
	Dist	Prox	Dist	Prox	Dist	Prox	Dist	Prox	Dist	Prox	Distal	Prox	Dist	Prox
Abd El-Moneum et al. [77]	50	50	51	51	35 (70%)	36 (72%)			11 (22%)	10 (20%)	30 (60%)	29 (58%)	11 (22%)	10 (20%)
Acar et al. [25]	350	350	61	62	213 (61%)	190 (54%)	215 (61%)	198 (57%)	126 (36%)	123 (35%)	151 (43%)	140 (40%)	124 (35%)	118 (34%)
Achim et al. [23]	44	44	63	65	32 (73%)	27 (61%)	44 (100%)	44 (100%)	21 (48%)	24 (55%)	40 (91%)	40 (91%)	22 (50%)	19 (43%)
Al-Azizi et al., [49]	150	150	66	67	119 (79%)	107 (71%)	94 (63%)	46 (31%)	51 (34%)	45 (30%)	27 (18%)	29 (19%)		
Amin et al., [50]	50	50	50	50	35 (70%)	35 (70%)	30 (60%)	30 (60%)	30 (60%)	30 (60%)			32,5 (65%)	32,5 (65%)
Aminian et al. [40]	650	657	68	68	479 (74%)	468 (71%)	237 (36%)	245 (37%)	196 (30%)	190 (29%)	442 (68%)	472 (72%)	126 (19%)	121 (18%)
Aoi, et al. [34]	202	206	69	69	131 (65%)	129 (63%)	90 (45%)	92 (45%)	76 (38%)	94 (46%)	169 (84%)	189 (92%)	16 (8%)	52 (25%)
Berezhnoi et al., [51]	84	57	82	82	27 (32%)	18 (32%)	84 (100%)	57 (100%)						
Chen et al., [76]	398	403	66	67	223 (56%)	227 (56%)	283 (71%)	120 (30%)	75 (19%)	82 (20%)	14 (4%)	16 (4%)	109 (27%)	128 (32%)
Chugh, et al. [7]	263	282	55	54	184 (70%)	198 (70%)	49 (19%)	44 (16%)	37 (14%)	37 (13%)			156 (59%)	200 (71%)
Dadarwal et al. [75]	160	160					42 (26%)	39 (24%)						
Daralammouri et al. [52]	104	105	56	59	77 (74%)	78 (74%)	27 (26%)	39 (37%)	43 (41%)	47 (45%)	12 (12%)	13 (12%)	50 (48%)	51 (49%)
Eid-Lidt et al. [74]	140	142	63	61	105 (75%)	109 (77%)	51 (36%)	49 (35%)	72 (51%)	62 (44%)	56 (40%)	55 (39%)	29 (21%)	24 (17%)
Elbayoumi et al. [73]	140	188					140 (100%)	188 (100%)						
Erdem et al. [8]	70	63	68	66	44 (63%)	44 (70%)	45 (64%)	50 (79%)	22 (31%)	22 (35%)	63 (90%)	58 (92%)	36 (51%)	35 (56%)
Feng et al. [53]	527	586	66	66	389 (74%)	423 (72%)	389 (74%)	197 (34%)	206 (39%)	214 (37%)	202 (38%)	253 (43%)	315 (60%)	364 (62%)
Gupta et al. [55]	210	210	55	54	126 (60%)	122 (58%)			66 (31%)	59 (28%)	93 (44%)	88 (42%)	118 (56%)	114 (54%)
Hammami et al. [72]	82	95	59	60	62 (76%)	70 (74%)	28 (34%)	36 (38%)	37 (45%)	38 (40%)			34 (41%)	40 (42%)
Kaledin et al. [20]	2775	3009												
Koledinskiy et al. [38]	132	132					132 (100%)	132 (100%)						
Korotkikh et al. [56]	391	385	63	63	242 (62%)	254 (66%)	164 (42%)	189 (49%)	102 (26%)	102 (26%)			110 (28%)	119 (31%)
Koutouzis et al. [11]	100	100	64	63	74 (74%)	77 (77%)	0 (0%)	0 (0%)	27 (27%)	28 (28%)	71 (71%)	59 (59%)	35 (35%)	28 (28%)
Koziński et al. [46]	200	200	67	67	120 (60%)	121 (60%)	123 (62%)	77 (38%)	64 (32%)	67 (34%)	132 (66%)	125 (62%)	93 (46%)	89 (44%)
Lee et al. [71]	42	26	60	59	30 (71%)	24 (92%)	42 (100%)	26 (100%)	8 (19%)	6 (23%)	8 (19%)	11 (42%)	15 (36%)	12 (46%)
Li et al. [57]	135	135	64	65	103 (76%)	107 (79%)	0 (0%)	135 (100%)	45 (33%)	40 (30%)			78 (58%)	84 (62%)
Li, Zhang et al. [21]	29	38	75	74	15 (52%)	21 (55%)	13 (45%)	14 (37%)	5 (17%)	6 (16%)	1 (3%)	0 (0%)	9 (31%)	9 (24%)
Lin, Sun et al. [78]	450	450	55	59	205 (46%)	225 (50%)	226 (50%)	216 (48%)	48 (11%)	56 (12%)			124 (28%)	101 (22%)
Lu et al. [58]	40	40	54	56	23 (57%)	25 (62%)	0 (0%)	0 (0%)	6 (15%)	7 (18%)				
Lucreziotti et al. [70]	100	104												
Noamen et al. [59]	125	12	64	64	83 (66%)	86 (69%)	101 (81%)	24 (19%)	59 (47%)	64 (51%)	51 (41%)	54 (43%)	32 (26%)	23 (18%)
Özkan et al. [69]	20	20												
Pacchioni et al. [68]	213	213	68	69	165 (77%)	164 (77%)	114 (54%)	112 (53%)	41 (19%)	36 (17%)	117 (55%)	121 (57%)	57 (27%)	59 (28%)
Pan et al. [67]	52	61	61	59	32 (62%)	36 (59%)	15 (29%)	20 (33%)	8 (15%)	8 (13%)			22 (42%)	19 (31%)
Roghani-Dehkordi et al. [66]	70	56	55	57	44 (63%)	36 (64%)	35 (50%)	20 (36%)	52 (74%)	45 (80%)	22 (31%)	16 (29%)	47 (67%)	44 (79%)
Ruzsa et al. [65]	38	157	69	67	26 (68%)	101 (64%)			18 (47%)	84 (54%)			7 (18%)	25 (16%)
Seecheran et al. [64]	88	23	60	59	61 (69%)	16 (70%)	20 (23%)	3 (13%)	53 (60%)	17 (74%)	22 (25%)	8 (35%)	22 (25%)	5 (22%)
Sharma et al. [63]	485	485	55	55	290 (60%)	285 (59%)								
Tehrani et al. [24]	33	31	66	69	23 (70%)	22 (71%)	20 (61%)	11 (35%)	12 (36%)	11 (35%)	25 (76%)	29 (94%)		
Tsigkas, et al. [17]	518	524	66	66	395 (76%)	396 (76%)	138 (27%)	118 (23%)	152 (29%)	158 (30%)	293 (57%)	264 (50%)	168 (32%)	169 (32%)
Tu et al. [62]	56	54	54	51	38 (68%)	36 (67%)	0 (0%)	54 (100%)	20 (36%)	22 (41%)	18 (32%)	22 (41%)	37 (66%)	39 (72%)
Wang, Liu et al., [79]	100	100	62	61	87 (87%)	80 (80%)	0 (0%)	100 (100%)	26 (26%)	18 (18%)	31 (31%)	33 (33%)	56 (56%)	52 (52%)
Wang, Peng et al. [61]	312	308	50	51	160 (51%)	157 (51%)			98 (31%)	87 (28%)	138 (44%)	129 (42%)	176 (56%)	168 (55%)
Wang, Yang et al. [37]	70	70	66	66	33 (47%)	35 (50%)								
Xu, et al. [60]	151	151	60	60	107 (71%)	95 (63%)	98 (65%)	132 (87%)	54 (36%)	55 (36%)	67 (44%)	60 (40%)	52 (34%)	50 (33%)

*dist*. Distal radial, *prox.* Proximal radial access group, *DM* Diabetes Mellitus, *HLP* Hyperlipidemia, *n* number of patients, *PCI* Percutaneous Coronary Intervention, *RCT* randomized controlled trial

### Primary endpoint: radial artery occlusion

**Fig. 2:**
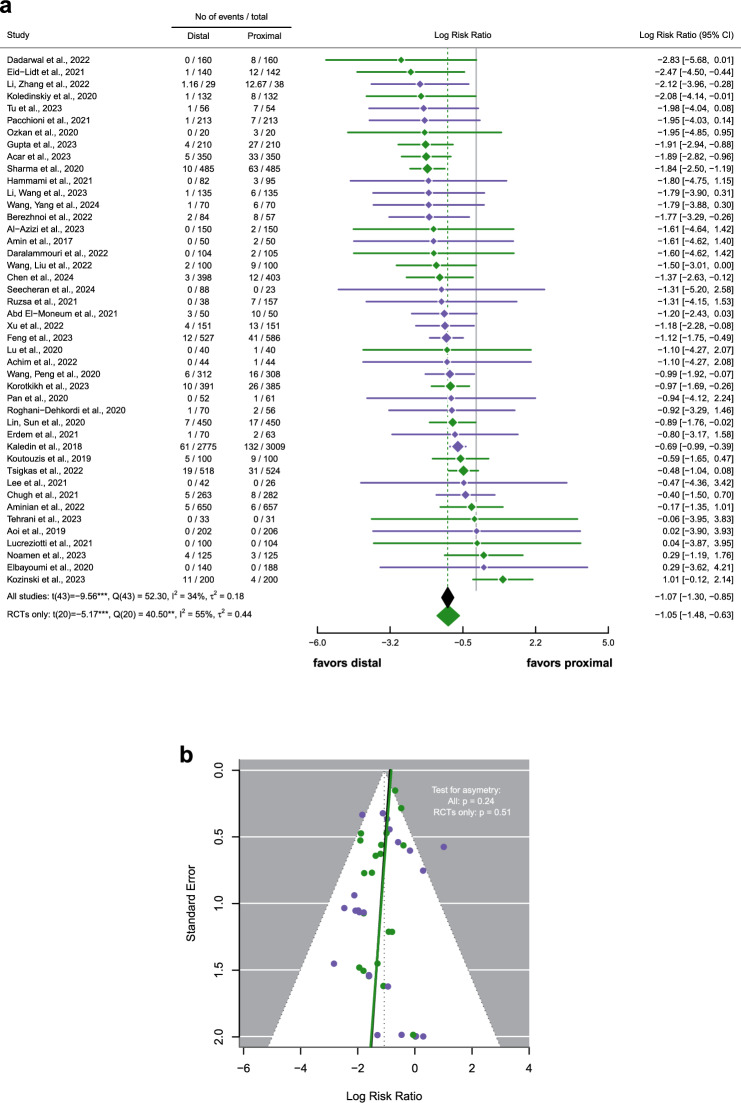
Meta-analytical results for the occurrence of RAOs (*k* = 37). RCTs are shown in green, non-RCTs in purple. **a** Forest plot for log risk ratio **b** Funnel plot including regression lines for the detection of possible publication bias †p < .10, *p < .05, **p < .01, ***p < .001

DRA showed a significantly lower rate of RAO compared to PRA. We found an incidence of RAO of 1.28% (95% CI: 0.97%–1.58%) for DRA and of 4.76% (95% CI: 3.47%–6.05%) for PRA. Thus, DRA had a 2.92-times lower risk than PRA for the occurrence of RAO (Log Risk Ratio = −1.07, *p* < 0.001, Fig. 2a). We did not detect significant heterogeneity (*Q* (43) = 52.30, *p* = 0.157) between the results. This finding was accompanied by a low relative effect heterogeneity (*I*^*2*^ = 34%). The funnel plot did not indicate publication bias visually, supported by a non-significant regression slope in the funnel plot (*b* = -0. 87, *p* = 0.242) (Fig. 2b). Selective analysis of the RCT-only subgroup did not alter any of the results.

### Secondary endpoints

**Fig. 3:**
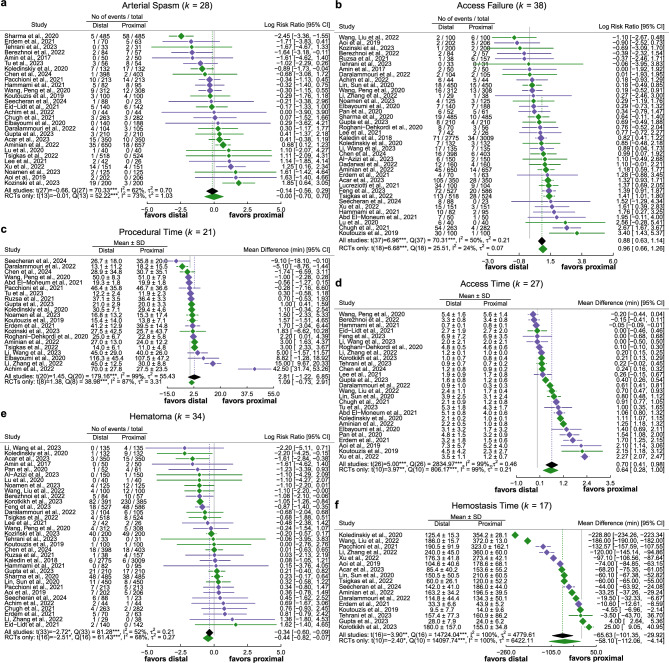
Meta-analytical results for the occurrence of Arterial Spasm (*k* = 28), Access failure (*k* = 38), Access time (*k* = 27), Procedural time (*k* = 21), Hematoma (*k* = 34), Hemostasis Time (*k* = 17). RCTs are shown in green, non-RCTs in purple. †p < .10, *p < .05, **p < .01, ***p < .001

Forest plots for all secondary endpoints are shown in Fig. 3. Only the results of moderator analyses that are statistically significant are reported.

#### Access failure

**Fig. 4:**
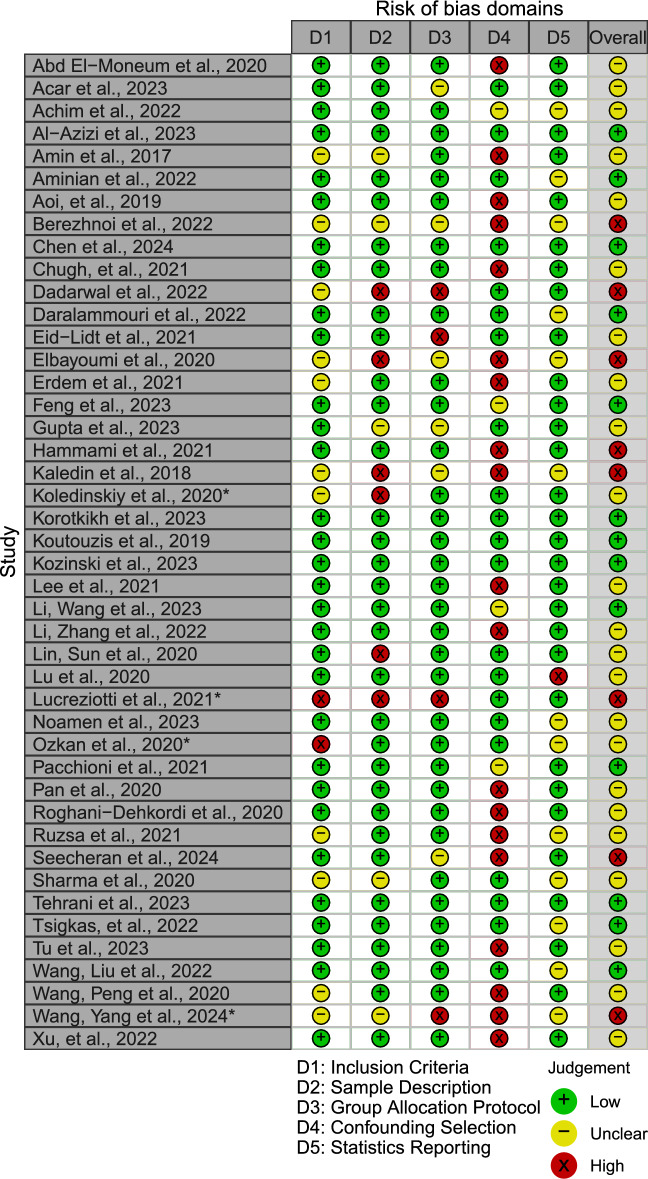
Risk of Bias rating included studies for transparency of inclusion criteria, sample description, group allocation (and protocol deviances), bias due to confounding and completeness of result reporting. *Studies for which only limited information from (conference) abstracts were available

In the 38 studies that provided data on successful catheterizations and crossovers, the risk for access failure was 2.42 times higher for DRA compared to PRA (Log Risk Ratio = 0.88, p < 0.001) (Figs. 3b, 4**)**.

The enhanced risk for access failure in the DRA group was most pronounced in studies with a low proportion of PCIs (b = −1.42, p = 0.009), while studies with > 80% PCI showed no difference in the rate of access failure between groups. Furthermore, differences were found for patients’ sex: differences in access failure were more pronounced in studies with higher proportion of males (b = 2.67, p = 0.046). This translated into an estimated risk ratio of 1.60 for studies with < 50% males, but an aggravated risk ratio of 2.71 in favor of PRA for studies with > 70% males. The aggregated results of the RCTs concurred for both effects.

#### Access time

Across studies, access time was heterogeneously defined, with some studies defining it as “puncture time” [22] and others defining it as “time to sheath insertion” [16], while still others did not define it at all, limiting the comparability of the studies*.* In the 27 studies that provided data on access time, the average access time varied between less than a minute and over five minutes. Proximal access was achieved 42 s faster on average than distal access (*p* < 0.001 for all studies, *p* = 0.003 for RCTs only, Fig. 3d).

#### Procedural time

Procedural time was on average 2 min, 49 s longer after DRA than after PRA, yielding a non-significant difference in 21 reporting studies (p = 0.162, Fig. 3c). One study reported a procedure time twice as long for DRA [23]. In the subset of RCTs, procedure time for PRA was non-significantly 65 s shorter than for DRA (p = 0.205).

#### Arterial spasm

Arterial spasms were 1.15 times more likely to occur in the PRA- than in the DRA group. However, no statistically significant difference with respect to the occurrence of arterial spasms was found (k = 28, p = 0.517; k_RCT_ = 14, p = 0.992, Fig. 3a).

#### Hematoma

34 studies reported on hematomas on the puncture site. Hematoma occurred significantly less often in DRA (−34% risk, p = 0.010). This effect was more pronounced in the RCT subgroup alone (−44% risk, k_RCT_ = 17, p = 0.002, Fig. 3e).

#### Hemostasis time

In the 17 studies reporting on hemostasis time, hemostasis occurred on average 66 min faster after DRA (p = 0.001, Fig. 3f). This finding was replicated for the subset of RCTs with a slightly attenuated difference of 58 min (k_RCT_ = 11, p = 0.037).

#### Crossover

In the 19 studies that provided information on the number and type of crossovers, there was a total of 270 crossovers from DRA to PRA, driven by a high number of 113 crossovers to PRA in the study by Tsigkas et al. [17]. There were 35 crossovers from DRA to femoral, 4 from DRA to ulnar, 3 from DRA to contralateral DRA, 8 from PRA to the contralateral PRA, 33 from PRA to femoral, 4 from PRA to ulnar and 7 from PRA to DRA. Two studies described 20 crossovers in the proximal and 56 crossovers in the distal group without naming the destination of the crossover [24, 25].

## Discussion

Using DRA as an alternative to PRA in clinical practice is currently highly debated [26, 27]. Proponents of the strategy argue that the significant reduction in RAO is the most relevant benefit for patients since it preserves the proximal radial artery for future interventions [28]. Shorter hemostasis time, as well as reduction of arterial spasm or hematoma may support this argument [29]. Still, concerns about longer procedure time and higher rates of access failure due to the more complex puncture remain [30]. Therefore, the use of DRA in the setting of acute coronary angiography or in more complex procedures is also discussed [30].

This meta-analysis makes a significant contribution to this debate by providing a comprehensive comparative overview of the advantages and disadvantages of DRA and PRA and thus offers a solid basis for evaluating DRA as a serious alternative to PRA. Our findings are: (1) the RAO-rate is significantly lower in DRA compared to PRA (p < 0.001), thus confirming that DRA provides a key advantage for this clinically relevant complication. However, our analysis also reveals that (2) access failure after DRA is significantly higher than after PRA (p < 0.001), suggesting that some reluctance to boost DRA as an equivalent alternative to PRA might be warranted.

In contrast to most comparable meta-analyses, which focus primarily on failure rate [31–33], our meta-analysis primarily considers the incidence of RAO, which we selected as the most clinically relevant endpoint for patients. In comparison, the failure rate is a more conditional parameter that is influenced by the experience of the interventionalist and could therefore be improved with increasing experience. The meta-analysis includes the largest set of studies that compare DRA to PRA to date as well as the highest number of RCTs. In comparison to Mufarrih et al. who analyzed the results of 8205 patients, we included a total of 21,081 patients in our meta-analysis [28]. Our analysis extends beyond the scope of existing meta-analyses by including the rate of access failure, access time, and the overall procedure time as further endpoints. In addition, possible clinical confounders were investigated for the first time using a moderator analysis. We discuss the clinical significance of the individual results in more detail below.

### RAO

Regarding the risk for RAO, we see a significant difference between the two approaches, with a 3.10 times lower risk (p < 0.001) for DRA. This result is consistent with the findings of nearly all meta-analyses published on this topic to date [28, 31–33]. In general, for both proximal and distal access, incidence of RAO was low, with several studies reporting not a single case [7, 23]. Nevertheless, in regard to the grave consequences for subsequent catheterization, the difference in RAO incidence is substantial. The preservation of the radial artery is essential, given that with increasing life expectancy of the population, we can anticipate an increase in the number of coronary interventions that patients will require. Given the irreversible nature of this complication, its prevention should be a primary objective in everyday clinical practice.

With an incidence of 1.30% RAO after DRA and 5.16% after PRA our results are within the range of 0.0–5.2% described previously [32, 33]. Mufarrih et al. confirm our result in their meta-analysis, evaluating only RCTs [28]. In their study from 2024, Lee et al. further corroborate the low incidence of RAO in a large representative cohort of 4,977 patients undergoing DRA [29].

One possible explanation for the significantly reduced rate of RAO after DRA is the location of the access point distal from the bifurcation of the radial artery into the deep palmar arch. Hence, upon puncture, the vascular network of the deep and superficial arterial branches may be more effective in preserving a sufficient blood flow in the radial artery [17, 34]. This effect might be amplified when there are longer procedures, multiple material changes and especially larger bore vascular catheters in the case of coronary interventions.

### Access failure

Our meta-analysis revealed a 2.44 times higher risk for access failure after DRA (p < 0.001), although other recent meta-analyses did not show a significant difference for access success between PRA and DRA [31, 35].

Greater difficulty in access could be explained by the smaller size of the radial artery in the anatomical snuffbox compared to the distal forearm [32]. Chugh et al. show a correlation between puncture time and access success with the diameter of the radial artery [7]. Consequently, access success was lowest in patients with DRA and a radial artery diameter < 1.6 mm [7]. Furthermore, the tortuous course of the distal radial artery poses a possible risk for difficult insertion of the wire after successful puncture [17]. Murai et al. developed an additional option to enhance the access success by utilizing a nitroglycerin patch prior to puncturing of the distal radial artery [36]. This significantly increased the success rate on the first attempt and also significantly reduced the number of puncture attempts needed [36]. The higher rate of successful access observed in studies with a high fraction of PCIs may be attributed to the greater puncture experience of interventionalists in the context of more complex interventions. In line with this, a certain learning effect related to distal puncture was described, increasing the access success rate over time [9].

### Procedural pain

In their meta-analysis, Mufarrih et al. also describe higher procedural pain as a potential consequence of significantly higher number of puncture attempts after DRA [28]. Access failure is not discussed in detail. In our meta-analysis, we concentrated on access failure as the parameter of procedural relevance in everyday life and a potential causal factor for procedural pain. One possible explanation for the more frequent occurrence of procedural pain after DRA could be the reduced application of local anesthetics at the distal access site. Still, Sgueglia et al. demonstrated with their RATATOUILLE study, published in 2022, that there was no deterioration of hand function following DRA in the 12-month follow-up period [3]. In their KODRA trial, Lee et al. found that 0.1% of all patients (6 of 4440 patients) experience hand dysfunction in the one-month follow-up, resulting in reduced mobility and painful movements of the thumb [29]. Thus, although the post-procedural pain appears to be stronger after the distal approach, it has not yet been associated with any lasting impairment of hand function. Implementing strategies to decrease access failure over time could therefore be an effective measure to reduce post-procedural pain.

### Access- and procedural time

As previously stated, the results indicating a faster access time following PRA must be interpreted with caution, given the heterogeneity of the various time definitions. Some studies stand out here, due to the establishment of specific protocols for distal access only. For example, in contrast to all other included studies that show similar procedure times, Achim et al. report the procedure time after DRA as twice as long [23]. Here, a specific hemostasis protocol was performed exclusively for DRA cases, and it is not specified whether this was included in procedural time. Excluding the data of Achim et al. decreased the time difference to 1 min and 36 s, yet the results remained non-significant (p = 0.106).

Longer access and procedure times could be considered a disadvantage of DRA, particularly in the context of urgent coronary angiography for myocardial infarction or more complex interventions such as in chronic total occlusion (CTO). Erdem et al. investigated the use of DRA in acute coronary syndromes and demonstrated that despite a longer sheath insertion time, there was no significant difference in procedural time for CAG (DRA 25.6 ± 4.2 min vs PRA 24.0 ± 5.7 min, p = 0.342) or PCI (DRA 44.3 ± 7.6 min vs PRA 43.5 ± 8.9 min, p = 0.421) in the setting of unstable angina pectoris, STEMI or NSTEMI [8]. These results were confirmed in the study by Wang et al. which reports no significant difference of puncture success rate and puncture time in the setting of STEMI [37]. Still, larger randomized trials are needed to examine the advantages and disadvantages of using DRA in an acute care setting. One study also showed DRA to be non-inferior to PRA for treatment of CTO [10]. In our meta-analysis, which included data from over 21,000 patients, the difference in access and procedural time between the distal and proximal approach was less than one minute. This finding suggests that with an increasing learning curve, the distal approach has no relevant disadvantages with regards to access and procedural time.

### Hemostasis time

Hemostasis occurred 66 min faster in DRA than in PRA (58 min in RCTs). In this context, Koledinsky et al. [38] is an outlier, since they reported a hemostasis time of 354.2 ± 28.1 min in the proximal against 125.4 ± 15.3 min in the distal group (p < 0.001). When excluding the data by Koledinsky et al., hemostasis time difference was 56 min and therefore still significantly shorter after DRA (p = 0.002). The shorter hemostasis time after DRA allows for a more rapid and complete mobilization and discharge of patients following coronary angiography through DRA. In addition, the shorter compression of the radial artery may causally contribute to the lower rate of RAO after DRA [34]. Optimal hemostasis using pulse oximetry to evaluate adequate patency is a known option to reduce RAO [39]. However, this technique is not widely used in the included studies, therefore opening the possibility of confounding [40]. There is currently no established, standardized procedure for hemostasis after DRA. Lee et al. describe various currently available options in the KODRA trial [29]. In addition, DRA-specific hemostatic devices are already being developed [41]. The current lack of standardization of the devices could be a contributing factor to the limited use of distal access in everyday clinical practice at this time.

### Crossover

Crossovers from distal to proximal radial approach occurred relatively often. This is in line with the higher rate of access failure in DRA.

In addition, we found 35 crossovers (13%) from DRA to femoral and 33 (12%) from PRA to femoral. The high number of crossovers to femoral is surprising, considering that PRA has been recommended over the use of femoral access since 2015 [42]. In the case of DRA, this may be based on the assumption that proximal puncture may also be difficult after failed puncture of the distal artery and therefore there is an increased risk of permanently damaging the radial artery.

### DRA in specific patient groups

With the increasing use of distal access, the number of clinical studies about DRA is also increasing, enabling a more detailed analysis of specific patient groups. Using the DISTRACTION registry, Oliveira et al. analyzed only patients older than 65 years who had undergone puncture of the distal radial artery [43]. Despite the presence of more complex coronary lesions and a greater number of multimorbid patients, the distal approach was found to be a safe procedure in this patient group. All studies included in this meta-analysis included more men than women, with an overall proportion of 66% men in all the studies analyzed [44]. Rivera et al. described a higher rate of arterial spasm after DRA in women [45]. In the study of Kozinski et al. female sex was significantly related to the primary composite endpoint of access crossover, major adverse cardiovascular events (MACE) and access-related vascular complications [46]. At the same time, the study describes a successful puncture in 99% of cases [46]. In our meta-analysis we found an increased incidence of access failure after DRA in studies with a high proportion of men, suggesting a higher risk for access failure in men after DRA. Thus, in order to identify sex-specific advantages and disadvantages in the use of DRA and PRA, more large, randomized studies are required.

### Current status in everyday clinical practice

Despite a substantial body of scientific evidence indicating the safety and feasibility of the distal radial approach, its routine clinical implementation remains limited [28, 31–33]. The more complex puncture with a higher rate of access failure appears to outweigh the clinical advantages of the access route. More complex positioning of the patient and more difficult position of the interventionalist are also discussed as potential factors [47]. Another potential explanation is the still limited availability of standardized hemostasis devices. Nonetheless, it is imperative that the current state of research be integrated into daily practice in order to benefit patients and that the medical industry and interventionalists continue to consider the further establishment of distal access [48].

## Conclusions

This meta-analysis demonstrates that DRA can be considered a feasible and safe access route for coronary angiographic procedures. With the inclusion of over 21,000 patients, this represents the most comprehensive meta-analysis of the distal approach to date, offering a detailed examination of the current advantages and disadvantages of DRA in comparison to PRA. One key advantage of DRA is the significantly reduced rate of RAO as well as a considerably shorter hemostasis time and no increase in arterial spasm or hematoma. The higher rate of access failure in addition to the longer procedure times, currently represent disadvantages compared to PRA. These maybe be contributing factors to the lack of widespread implementation of DRA at this time. Nevertheless, it is likely that these disadvantages will become less pronounced as more experience with DRA is gained by interventionalists. It is recommended that further large, randomized trials be conducted in order to provide more generalized recommendations on DRA as an alternative standard access route in angiography. These trials should take individual factors such as sex and type of intervention into account.

## Limitations

This meta-analysis does not report on long term cardiovascular outcomes, including secondary myocardial infarctions or cerebrovascular events. Medium- and long-term side effects at the access or detailed information about hand function are not included into this analysis. In comparison to Mufarrih et al., we included not only randomized controlled trials, but also observational studies and registries in our analysis. However, this enabled us to analyze a large, representative patient clientele in order to generate reliable and transferable data. Important limitations derive from a heterogeneous definition of the secondary endpoints analyzed in this meta-analysis. This includes access and procedural time, as well as the number and type of crossovers. Therefore, it was not possible to make a generalizable comparison regarding those endpoints since the available data was both highly heterogenous and lacked standardization. It is recommended that this be taken into account in future studies, with the aim of establishing a uniform and standardized definition of the endpoints under investigation.

## Supplementary Information

Below is the link to the electronic supplementary material.

Supplementary file1 (PDF 187 KB)

## Data Availability

All data analysed in this study are available from the corresponding author upon reasonable request.
