# Supplementary material for: Distal versus proximal radial access in coronary angiography: a meta-analysis
Source: Clin Res Cardiol. 2024 Sep 17;115(1):1–15. doi: 10.1007/s00392-024-02505-3 (PMC12783226; doi:10.1007/s00392-024-02505-3)
Supplement: Supplementary file 1 — Supplementary file1 (PDF 187 KB) [file 392_2024_2505_MOESM1_ESM.pdf]

Supplementary Data

	Low RoB	Some concerns	High RoB
**Inclusion Criteria**	Defined: Who, when, underlying disease, purpose of the examination (CAG/PCI)	Something remains unclear	Not specified
**Sample Description**	Precisely named (demographic and clinical information)	Partially complete	Missing
**Group Allocation (Protocol)**	Transparent who is in which group and whether and how changes occured ("cross-overs")	Minor ambiguities, but comprehensible	Unclear sample sizes
**Confounding**	Randomised	Matching or using covariates with non-randomised data	Non-randomised data without any statistical bias correction
**Statistics**	Appropriate methods and presentation of results (comprehensible/complete)	Deficiencies/ ambiguities/ inaccuracies	Lack of comprehensibility (e.g. only p-value without description)

Supplementary Table 1 Risk of Bias Assessment

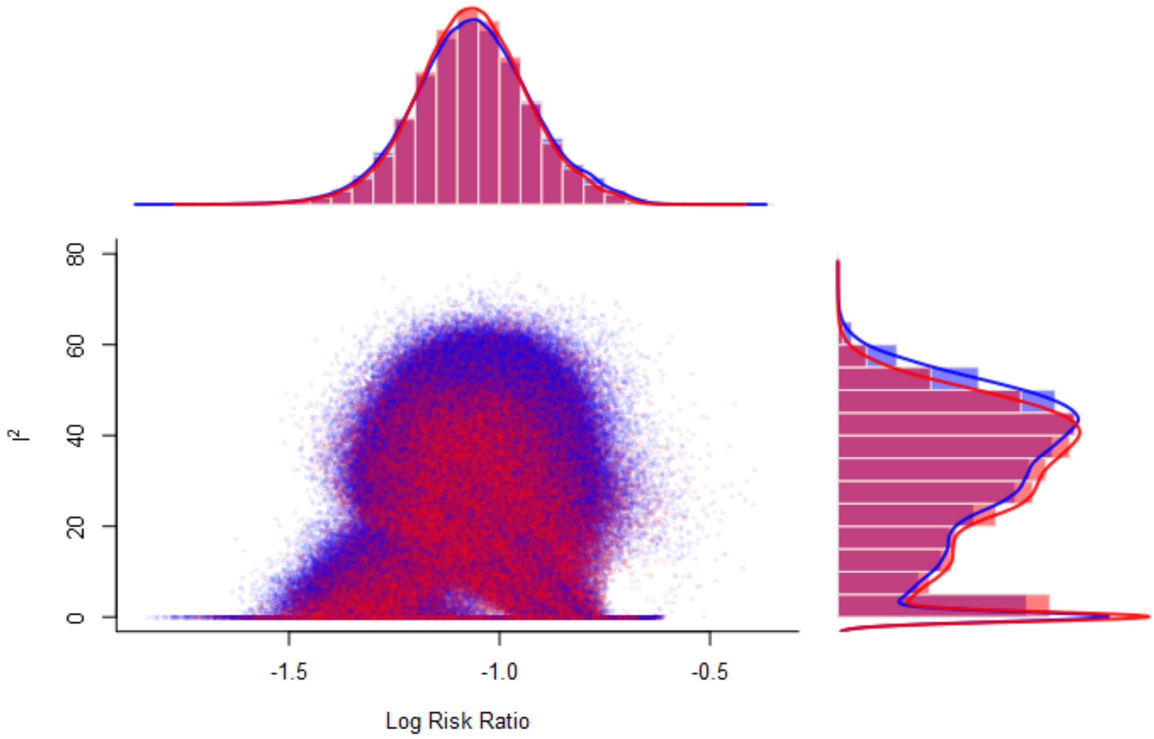

Supplementary Figure 1 Study heterogeneity was studied via a GOSH plot [44]

To this end, a Baujat plot (not shown) representing our main analysis (RAO) was plotted in order to identify studies that represent outliers in comparison to the bulk of other studies. Kozinsky et al. was identified as the sole outlier; not surprisingly, as this study reported the strongest evidence in terms of RAO in favor of PRA [43]. The according GOSH plot is shown in Supplementary Figure 1. This plot emphasizes a) that no substantial heterogeneity between studies could be observed (as indicated by the fuzzy outline of the scatter plot, only skewed at the bottom by those studies that reported no RAOs at all), and b) that the contradictory finding by Kozinski et al. does not bias the aggregated RAO risk, as this study's contribution is again distributed rather homogeneously in all possible subsets of studies (shown in red).
